# Psychosocial profiles of physical activity fluctuation in office employees: A latent profile analysis

**DOI:** 10.1371/journal.pone.0227182

**Published:** 2020-01-08

**Authors:** Yanping Duan, Borui Shang, Wei Liang, Min Yang, Walter Brehm

**Affiliations:** 1 Department of Sport and Physical Education, Hong Kong Baptist University, Hong Kong, China; 2 Department of Kinesiology, Hebei Sport University, Shijiazhuang, China; 3 Graduate School, Wuhan Sports University, Wuhan, China; 4 Institute of Sports Science, University of Bayreuth, Bayreuth, Germany; Qazvin University of Medical Sciences, ISLAMIC REPUBLIC OF IRAN

## Abstract

**Objectives:**

Fluctuation is a common but neglected phenomenon of physical activity (PA) behavior. This study aimed to explore the psychosocial profiles of PA fluctuation in office employees, and to examine the association of latent profiles with demographics and PA level.

**Method:**

434 Chinese office employees who were identified as PA fluctuators (*M* = 32.4 years, *SD* = 6.9, 55.5% female) completed a cross-sectional online survey covering demographics, PA behavior, and six psychosocial indicators (self-efficacy, planning, action control, affective attitude, social support, and perceived barriers). Latent profile analysis was used to determine PA fluctuators’ psychosocial profiles. Associated factors of profile membership were identified with multinomial logistic regression.

**Results:**

The two-profile model (uncommitted vs. moderately committed) was selected as the best solution. The moderately committed group (n = 346, 79.7%) possessed a more active mindset by reporting significantly higher scores of self-efficacy (t = 9.42 p < .001), planning (t = 16.33 p < .001), action control (t = 14.55 p < .001), affective attitude (t = 13.33 p < .001), and social support (t = 11.50 p < .001) compared with the uncommitted group (n = 88, 20.3%). Results from a multinomial logistic regression showed that the moderately committed profile was associated with normal weight status (OR = 2.00, *p*< .05), having a medium managerial position (OR = 2.54, *p*< .01), and high level of moderate to vigorous PA behavior (OR = 4.85, *p*< .001).

**Conclusions:**

These findings demonstrate the variability of PA fluctuators’ mindsets. Future tailored interventions are recommended to promote PA behavior for this population based on the categorization from the present study.

## Introduction

It is well-established that regular and sufficient physical activity (PA) is associated with significant benefits for individual physical and mental health [1–4]. The World Health Organization formulated the widely acknowledged Health-Enhancing Physical Activity (HEPA) guidelines, suggesting that in order to receive these substantial health benefits, adults aged 18–64 years should accumulate at least 150 minutes of moderate-intensity PA per week, or 75 minutes of vigorous-intensity PA, or an equivalent combination [5, 6]. Despite the well-known benefits of HEPA, adherence to regular HEPA is still difficult for the majority of the population [7]. In particular, some people may adhere to the HEPA guideline over a period of time, and then decline to being physically inactive for another period of time, followed by a rebound to being physically active again [8]. The name specified for this particular group of people is “fluctuators”, and their fluctuating PA behavior pattern is labeled “fluctuation” [9].

PA fluctuators can often be observed among office employees [10]. The nature of work in office settings does not require strong physical effort, which results in sedentary behavior and a low level of PA during working hours [11]. In addition to this, research showed that the time during which office employees engaged in PA (light or moderate intensity) only accounted for 40% of their outside working hours [12], indicating that office employees execute PA occasionally and insufficiently during their leisure time. In this regard, literature has shown that the percentage of office employees classified as PA fluctuators ranges from 16% to 69% [10, 13], and this trend is increasing rapidly [11]. Such insufficient PA patterns are associated with negative health outcomes among office employees, including cardiovascular diseases, musculoskeletal disorders and other chronic diseases [14, 15]. In order to support fluctuators to engage in regular PA and receive health benefits, understanding fluctuators’ PA behavior and their related psychosocial features is a crucial prerequisite.

A recent exploratory review laid the foundation for PA fluctuation investigation by mapping definitions, measurements and empirical evidence [16]. In particular, this review summarized that PA fluctuation is: 1) primarily defined within the stage model constructs, which elaborate the progression from insufficient PA participation to habitual PA behavior [9, 17]; 2) measured accordingly by self-reported stage algorithms [18, 19]; and 3) demonstrated by stage distinctiveness among other PA stages [17, 20]. Especially regarding the stage distinctiveness of fluctuation, researchers commonly adopt the “inter-stage comparison” paradigm, which compares the differences between fluctuation stage and other PA stages in relation to certain psychosocial variables (e.g., self-efficacy, social support) during the stage transition.

While the existing “inter-stage comparison” paradigm described above is useful in its own right to explore the fluctuation, it cannot reflect the intra-stage differences or heterogeneity within fluctuators regarding their internal psychosocial profiles. Namely, it is still unclear whether there are distinct psychosocial profiles among sub-categories of fluctuators. The person-centered approach is well-suited for categorizing individuals according to their characteristics. From a statistical perspective, the person-centered approach and the variable-centered approach differ fundamentally in their assumptions about whether a sample of individuals is from a single population or sub-populations within a single population. The person-centered approach allows the possibility that the sample might reflect multiple sub-populations characterized by different sets of parameters, and *vice versa* for the variable-centered approach. The person-centered approach can be used to identify subgroups with different mindsets [21]. Latent profile analysis (LPA) as a person-centered approach can categorize individuals, and provides a more holistic picture of individuals, with complex interactions among multiple variables [22]. The LPA is model-based and several fit indices can be generated to compare among different models, and to help researchers making less arbitrary decisions regarding the optimal number of latent profiles [23]. There has recently been an increase in the LPA approach in the investigation of PA behavior [24–26]. To date, however, no study has applied LPA to identify subgroups of PA fluctuators.

To explore the profile membership of PA fluctuators, it is essential to identify the psychosocial indicators constituting a profile when LPA is conducted. It has been found that in general, PA fluctuators are characterized by three social-cognitive characteristics, including: 1) intention and readiness for PA participation; 2) low automaticity (PA participation is not habitual); 3) limited self-regulation [16]. All three of these characteristics fall into the post-intention/volitional phase described in the Health Action Process Approach (HAPA) [27]. The HAPA model posits five proximal psychosocial factors that are assumed to influence the PA behavior process during the volitional phase, including self-efficacy, planning, action control, perceived barriers, and social support [28]. Besides the five psychosocial factors in the HAPA, it has been suggested that affective attitude (constructs related to emotion and feelings derived from participating in PA) can also influence individuals' PA maintenance [29, 30].

Among the six aforementioned psychosocial factors, previous empirical studies have shown that five factors (self-efficacy, planning, perceived barriers, social support and affective attitude) can effectively distinguish the fluctuation stage from the preparation and maintenance stages [16]. In terms of action control, although there are no studies testing the differences in action control between fluctuation and other adjacent stages, it has been examined as a crucial construct in adherence to long-term plans of health behavior, and as a bridge that closes the intention-behavior gap [28]. Thus, it is assumed that action control can be associated with PA fluctuation.

In addition to using LPA to identify the profile membership of individuals, the person-centered approach suggests a further examination of the relationship between the profile membership and its correlates [21]. Since no previous research can be referred to for the examination of profile correlates, the correlates were selected based on those variables related to the stages of change for PA behavior (with fluctuation as one of the stages of change). It has been found that demographic variables (e.g., gender, Body Mass Index) and PA level are significantly associated with PA stages. People who are at higher PA stages (e.g., maintenance, exploration, fluctuation) are more often female, more often fall within the normal weight range, and expend more energy on PA compared to those at lower PA stages (e.g., preparation, contemplation) [20, 31].

With this background, the purpose of the current study is two-fold: first, to explore the heterogeneity of PA fluctuators’ psychosocial profiles by attempting to classify fluctuators into different sub-categories; second, to examine the association of membership with demographics and PA level. In light of the exploratory nature of this study, the following research questions were addressed:

1. What are the distinct sub-categories that best summarize the complexity of PA fluctuators, based on psychosocial indicators? Which psychosocial indicators differentiate the sub-categories from each other?
2. Are demographic variables and PA level associated with membership in different fluctuator sub-categories?

## Methods

### Study design, procedure, and participants

This study was a cross-sectional survey study. Ethical approval was granted by the research ethics committee of the Hong Kong Baptist University. Using convenience sampling, office-based employees between 18 and 60 years old were recruited from four types of organizations in China, including state-owned enterprise, private enterprise, educational organization (e.g., university or college), and the government sector. The personnel officers in charge of the organizations were first contacted. With their permission, a website hyperlink to the online survey was delivered to them, which included informed consent forms. Officers sent the online hyperlink to their employees via a smart phone group chat platform used only within their organization. The online survey was conducted through *Sojump–*a professional online data collection platform in China, which is similar to *Qualtrics* [32]. This platform allows the researcher to control the quality of responses by setting up a series of standards (e.g., minimum answering time, minimum response rate) and offering incentives for qualified responses. Prior to the beginning of the questionnaire survey, an informed consent page with details of the survey was presented to each participant. Participants were only able to start the survey once they click the agreement option.

In total, 1868 office-based employees were contacted online. Among them, 1746 responders participated in the online survey, resulting in a response rate of 93.5%. Questionnaires with the following characteristics were excluded: 1) missing data (over 50%; n = 25, 7.8%); 2) responses that had been provided too quickly (n = 89, 27.9%). The minimum reasonable answering time was set as 150 seconds, which was based on a pilot test of 20 participants, counting the average minimum answering time; 3) participants with a disease impeding normal PA (n = 69, 21.6%); 4) inappropriate occupation (not office work, n = 61, 19.1%); 5) inappropriate age (age under 18 or above 60, n = 17, 5.3%); and 6) other miscellaneous reasons (e.g. IP address of participant not in China, answering every question with the same choice; n = 58, 18.2%). As a result, 1427 (81.7%) valid questionnaires remained for analysis.

Using the stage algorithm [33], 434 out of 1427 (30.4%) participants were further identified as PA fluctuators. The mean age of fluctuators was 32.4 years (*SD* = 6.9) and ranged from 19 to 59 years, with 241 females (55.5%). The mean value of BMI of fluctuators was 22 (*SD = 3*.*2*) and ranged from 14.8 to 38.1.

### Measures

The measurement items consisted of four components covering demographics, stage algorithm, PA behavior and six psychosocial indicators.

#### Demographics

Participants were asked to provide demographic information regarding their age, gender, education level, job position, marital status, parental status, height (m) and weight (kg). Body Mass Index (BMI) was computed with the formula of kg/m^2^.

#### Stage algorithm

The stage algorithm of the FIT model is a self-report multiple-choice questionnaire with six statements aiming to categorize individuals into six stages according to their thoughts and behaviors regarding PA behavior [17]. The six stages are titled not-considering, considering, preparing, exploring, fluctuating and maintaining. This algorithm has been found to be a reliable and valid instrument in previous research among Chinese adults [20, 33]. In the present study, the PA criteria were set as “at least 150 minutes moderate to vigorous physical activity (MVPA) per week”, according to the WHO criteria [6]. The FIT stage algorithm was used in this study to select those from the entire sample of people that were in the fluctuation stage. If participants selected the fifth statement "I am physically active, but not regularly every week", or "have not accumulated at least 150 minutes every week", they were assigned to the fluctuation stage for further classification.

#### Godin-Shephard Leisure-Time Physical Activity Questionnaire (GSLTPAQ)

The GSLTPAQ was developed to assess leisure-time PA participation at three different intensities (mild, moderate, strenuous) [34]. The questionnaire has been translated into multiple languages, including Chinese, the Chinese version was found to have acceptable reliability and validity [35]. The overall weekly PA energy expenditure indicated by the Leisure Score Index (LSI) was calculated with the formula “LSI = (frequency of mild × 3) + (frequency of moderate × 5) + (frequency of strenuous × 9)” [36]. The cut-off values for the classification scoring were based on the North American public health PA guidelines. These guidelines state that LSI (only for moderate and strenuous) ≥ 24 is classified as active, whereas LSI (only for moderate and strenuous) ≤ 23 is classified as insufficiently active [37]. Previous research has shown that the mean minutes of moderate vigorous physical activity (MVPA) in adults classified as active by the GSLTPAQ (LSI ≥ 24) almost reach the recommended WHO recommended 150 minutes of MVPA/week, and the consistency between these two PA classification scores was found to be moderate (r = .46, *p* < .0001) [37].

#### Six psychosocial indicators

All questionnaires using the six psychosocial indicators have been well validated in previous studies among the Chinese adult population [17, 38, 39]. In addition, analysis of data from a pilot test targeting 73 office-based employees (male = 38, 52.1%; M = 31.0 yrs, SD = 7.6) showed acceptable reliability of the questionnaires.

*Self-efficacy* for PA was assessed by the stem “I am confident I can participate in regular PA (accumulated at least 150 minutes moderate or high intensity per week) even when…” followed by five items such as “…I am tired” (Cronbach's alpha = .85). Answers were given on a 5-point scale ranging from 1 “not at all true” to 5 “exactly true”.

*Planning* was assessed by the stem “For the next month I have planned…” followed by five items such as “…which concrete PA I will pursue” (Cronbach's alpha = .93). Answers were given on a 5-point scale ranging from 1 “not at all true” to 5 “exactly true”.

*Action control* was assessed with six items asking how people regulate their PA behaviors (Cronbach's alpha = 0.90), such as “Usually for my PA behavior, I have constantly monitored myself whether I exercise frequently enough”. Answers were given on a 4-point scale from 1 “not at all true” to 4 “exactly true”.

*Affective attitude* was assessed by the stem “When I am thinking of participating in PA, I will feel…” followed by four items including “…pleased”, “…satisfied”, “…happy”, and “…comfortable” (Cronbach's alpha = .96). Answers were given on a 7-point scale from 1 “not at all true” to 7 “exactly true”.

*Social support* was assessed with three items including “My partner helps me/my family members help me/my colleagues and friends help me to stay physically active (Cronbach's alpha = .82). Answers were given on a 5-point scale from 1 “not at all true” to 5 “exactly true”.

*Perceived barriers* were assessed with six items to inquire about the degree to which situations prevent people from doing PA, such as “Tight work schedule”, “Lack block of time” (Cronbach's alpha = .77). Answers were given on a 3-point scale from 1 “never”, 2 “sometimes” to 3 “very often”.

### Data analysis

Mplus 8 was used to conduct latent profile analysis (LPA) [40]. Models incorporating 1 to 6 profiles were estimated using robust maximum likelihood estimators. The means and variances of the six indicators were freely estimated in all profiles [41], using 300 random sets of starting values in the initial stage and the 20 best solutions retained for final stage optimization [42]. All models converged on well replicated solutions. LPA was performed with six psychosocial indicators (self-efficacy, planning, action control, affective attitude, social support, perceived barriers).

For the determination of the optimal number of profiles, both statistical adequacy and non-statistical substantive interpretation of the profiles should be considered [22]. For the test statistic values, Akaike information criterion (AIC), Bayesian information criterion (BIC), sample-size adjusted BIC (SABIC), LO-MENDELL-RUBIN likelihood ratio (LMR) and the Bootstrap Likelihood Ratio Test (BLRT) were reported. Noticeably, the entropy was also reported only to demonstrate the precision with which the cases were classified in the profiles (on a 0 to 1 scale). However, the entropy does not serve as a main indicator to determine the optimal number of profiles [43]. A lower value on the AIC, BIC and SABIC suggests a better-fitting model. Both the LMR and BLRT compare a k-profile model with a k-1-profile model. A significant p value indicates that the k-1-profile model should be rejected in favor of a k-profile model [44]. Since the information criteria (AIC, BIC, and SABIC) derived from the class enumeration procedure can be heavily influenced by sample size, these criteria frequently continue to increase with the addition of latent profiles without reaching a minimum value [22]. Given this, the statistical values of AIC, BIC and SABIC should be graphically presented in an elbow plot to illustrate the changes associated with additional profiles [45]. In the elbow plot, the point after which the slope starts to show the trend of flattening suggests the optimal number of profiles. After identifying the profile membership, one-way ANOVAs (for at least three profiles) or independent t-tests (for two profiles) were used to examine the discrepancies of profiles regarding psychosocial indicators.

Additionally, multinomial logistic regression analysis (MLRA) was applied to examine whether demographic and PA behavioral variables were associated with the profile membership of PA fluctuators. Demographic variables (e.g., gender, age, marital status, job position, BMI etc.) and the PA behavioral variable (energy expenditure) were regarded as independent variables in the regression model. The new auxiliary option of “R3STEP” in Mplus was applied. Rather than assigning a 100% probability that each individual belongs to a certain profile, “R3STEP” allows for measurement error (retaining the original probability of profile membership) in the independent evaluation of the relationship between the latent profile and the independent variables [46]. This method has been widely acknowledged as an advantageous approach over traditional methods of categorically assigning individuals to the most likely latent class [46].

## Results

### Descriptive statistics

Descriptive statistics of PA fluctuators’ characteristics and Leisure Score Index of PA are presented in Table 1. 96.1% (*n* = 417) of fluctuators had an education level equal to or higher than a bachelor degree. 59.8% (*n* = 259) of fluctuators were non-managerial office-based employees, while 40.2% (*n* = 174) held high or medium managerial positions. 71% (*n* = 308) of fluctuators were married and 62.9% (*n* = 273) of fluctuators had children. Most of the fluctuators (*n* = 326; 76.3%) were within a normal weight range, followed by weight status of being overweight (*n* = 65; 15.2%), and underweight (*n* = 36; 8.4%). Nearly half (*n* = 222; 51.2%) of the fluctuators were sufficiently active, while the other nearly half (*n* = 212; 48.8%) were insufficiently active. For the descriptive statistics of psychosocial indicators, mean scores for self-efficacy, planning, action control, affective attitude, social support and perceived barriers were 2.59 (SD = 0.79), 3.24 (SD = 0.85), 2.84 (SD = 0.63), 5.02 (SD = 1.34), 3.15 (SD = 0.95), and 2.00 (SD = 0.37), respectively. Since LPA has a relatively low demand on the data distribution and does not require normal distributions of indicators, the original scores of indicators were used rather than data transformations [23].

**Table 1:** *Sample characteristics and leisure score index (LSI) of physical activity (N = 425–434)*.

		N	%	M ± SD	Range
Age (years)		425		32.4 ± 6.9	19–59
	<30	168	39.5		
	30–39	201	47.3		
	≥40	56	13.2		
Gender		434			
	Male	193	44.5		
	Female	241	55.5		
Education		434			
	High school or below	17	3.9		
	Bachelor	334	77.0		
	Post-graduate or above	83	19.1		
Job position		433			
	Senior managerial position	19	4.4		
	Middle managerial position	155	35.8		
	Non-managerial staff	259	59.8		
Marital status		434			
	Married	308	71.0		
	Unmarried	126	29.0		
Parental status		434			
	Children	273	62.9		
	No children	161	37.1		
BMI^1^ (Kg/m^2^)		427		22.0 ± 3.2	14.8–38.1
	Underweight (BMI<18.5)	36	8.4		
	Normal weight (18.5≤BMI<25)	326	76.3		
	Overweight (BMI>25)	65	15.2		
Leisure score index (LSI)^2^		434		25.5 ± 15.7	0–98
	Sufficiently active (≥24)	222	51.2%		
	Insufficiently active (< 24)	212	48.8%		

Note.

1. BMI classification criteria are from World Health Organization (2004)

2. The LSI classification uses only moderate and strenuous scores. Those with a leisure score index ≥ 24 were classified as sufficiently active; those with a score ≤ 23 were classified as insufficiently active (Amireault & Godin, 2015).

### Latent profile analysis

**Fig 1:**
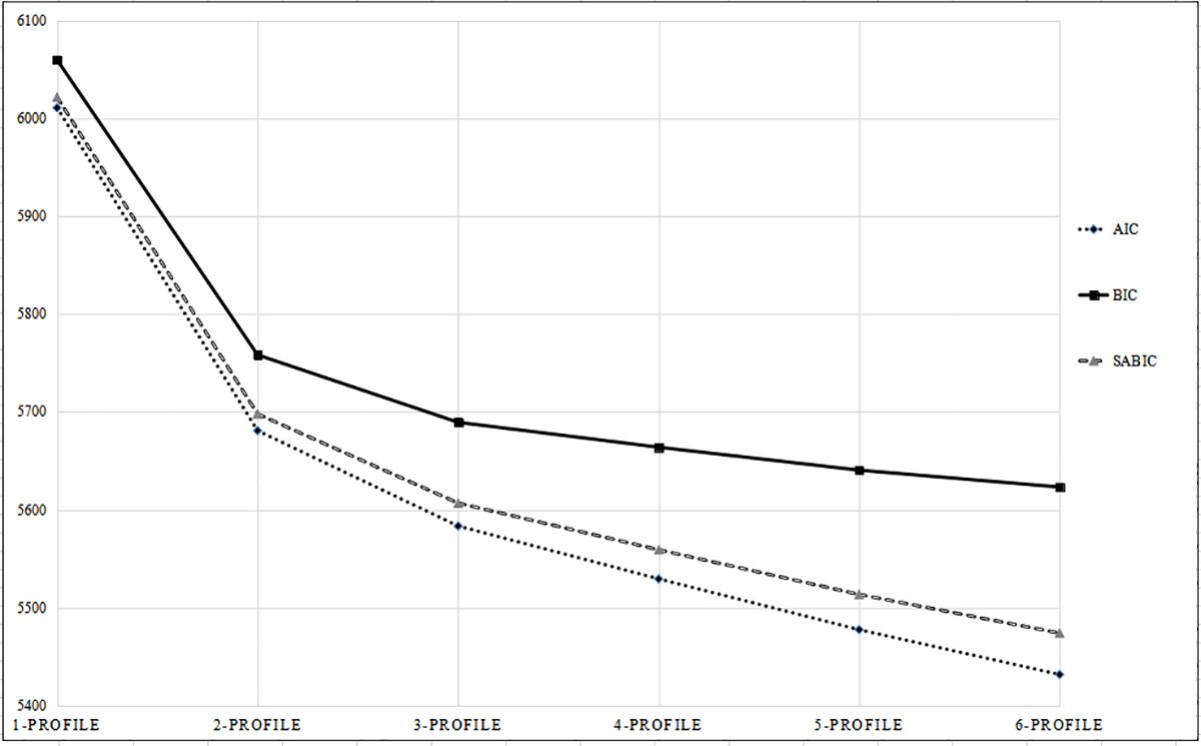
Elbow plot of the information criteria for the latent profile analysis.

Results of the LPA are presented in Table 2. For the three information indices (AIC, BIC, and SABIC), the values continued to decrease when the number of profiles increased. Comparisons of the AIC, BIC, and SABIC for different models were contrasted in an elbow plot (see Fig 1). As illustrated, the elbow plot suggests that the improvement in goodness of fit starts to show the trend of flattening with the two-profile model (AIC = 5680.768; BIC = 5758.156; SABIC = 5697.860). In other words, despite the improvement of model fit indices as the number of profiles increased, the marginal improvement became more negligible after reaching the two-profile model. In the current study, we stopped analyzing the models with more than six profiles, because a steady trend in the elbow plot had been established, indicating it was unlikely that we would find a better model thereafter.

**Table 2:** *Fit indices*, *entropy*, *and model comparisons for estimated latent profile analyses models*.

Models	Log-likelihood	AIC	BIC	SABIC	P value of LMR	P value of BLRT	Entropy	Number of people in each profile
One Profile	-2993.282	6010.56	6059.44	6021.36	/	/	/	434
Two Profiles	-2821.384	5680.77	5758.16	5697.86	< .001	< .001	.804	88, 346
Three Profiles	-2765.714	5583.43	5689.33	5606.82	.387	< .001	.803	76, 316, 42
Four Profiles	-2731.728	5529.46	5663.87	5559.14	.470	< .001	.807	60, 282, 18, 74
Five Profiles	-2698.771	5477.54	5640.47	5513.53	.232	< .001	.791	49, 172, 33, 174, 6
Six Profiles	-2668.888	5431.78	5623.21	5474.06	.250	< .001	.803	16, 55, 143, 182, 32, 6

Note.

AIC = Akaike Information Criterion; BIC = Bayesian Information Criterion; SABIC = Sample-size Adjusted BIC; LMR = LO-MENDELL-RUBIN likelihood ratio test; BLRT = Bootstrap Likelihood Ratio Test

Results based on the LMR and BLRT tests also supported the two-profile solution. Specifically, the LMR likelihood ratio tests only demonstrated significant results when comparing the two-profile model versus the default one-profile model (*p* < .001). The results of the BLRT demonstrated that the p- values were all significant (all *p* < .001), indicating that the model becomes better fit with the addition of the profile number. It was also shown that the entropy values were quite high (> .800) for all of the estimated models, with the exception of the five-profile model. Moreover, the two-profile model did not result in an extreme distribution of profiles (under 5% of either one of the profiles). In the current two-profile solution, the number of fluctuators in each were 88 (20.3%) and 346 (79.7%), avoiding the extreme distribution with profile membership of less than 5%. Given the results mentioned above, the two-profile model was finally chosen as the best representation of the data.

### Profile labeling and interpretation

**Fig 2:**
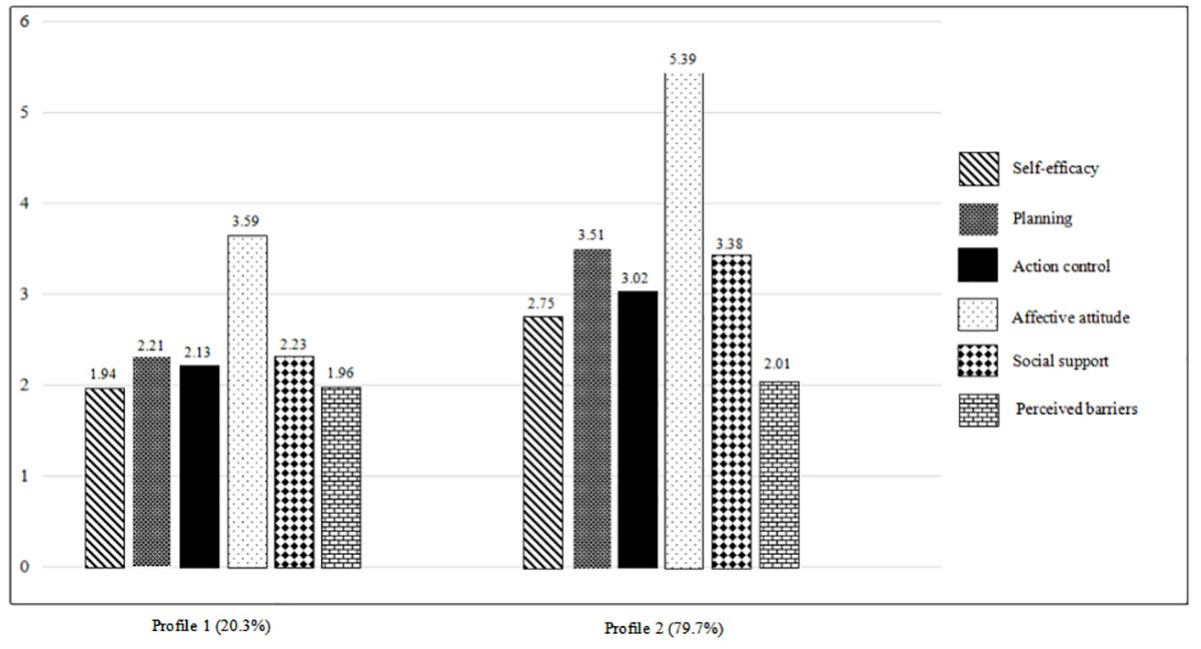
Characteristics of the latent profiles on all six psychosocial indicators.

Descriptive results of psychosocial indicators by profile are presented in Fig 2. Profile 1 (*n* = 88), which accounts for 20.3% (88/434) of the sample fluctuators, demonstrated comparatively lower scores on all six psychosocial indicators compared with those of profile 2 (*n* = 346), accounting for 79.7% (346/434) of the sample. When mean scores of psychosocial indicators between the two profiles were compared, it was found that except for perceived barriers (t = 1.10, *p* = . 272), all psychosocial indicators including self-efficacy (t = 9.42), planning (t = 16.33), action control (t = 14.55), affective attitude (t = 13.31) and social support (t = 11.50) were significantly different between the two profiles (all *p* < .001).

Given the aforementioned results, profile 1, with lower scores of psychosocial indicators, was labeled as “uncommitted” (UC) fluctuators, indicating that the fluctuators might not be psychosocially ready or committed to PA participation. Profile 2, on the other hand, with higher scores of psychosocial indicators, was labelled as "moderately committed (MC)" fluctuators.

### Multinomial logistic regression results

Table 3 presents the results of the MLRA, with the UC profile treated as the reference group. The regression model showed a significant relationship between profile membership of PA fluctuators and the selected predictors (*Chi*-square = 54.99, *p* < .001). As shown in Table 3, job position, weight status, and PA energy expenditure were all significant predictors of profile membership. Specifically, fluctuators in middle managerial positions were more likely than non-managerial fluctuators to be members of the MC profile (*OR* = 2.54, 95% *CI* = 1.35 to 4.77, *p* < .01). Moreover, compared with the overweight fluctuators (BMI ≥ 25), fluctuators with a normal weight status (18.5 ≤ BMI < 25) were significantly related to membership in the MC profile (*OR* = 2.00, 95% *CI* = 1.03 to 3.90, *p* < .05). As expected, being more active (LSI ≥ 24) was significantly related to the MC profile rather than the UC profile (*OR* = 4.85, 95% *CI* = 2.77 to 8.47, *p* < .001).

**Table 3:** *Results of multinomial regression for predicting profile membership (n = 434)*.

Name of variable	*OR*	95% *CI*
Age (≥40 years as reference group)		
30–39 years	1.14	0.53–2.45
≤ 29 years	1.04	0.43–2.51
Gender (female as reference group)		
Male	0.94	0.54–1.62
Education (post-graduate and above as reference group)		
Bachelor degree	1.48	0.80–2.73
High school diploma or below	2.20	0.51–9.42
Job position (Non-managerial staff as reference group)		
Middle managerial staff	2.54**	1.35–4.77
Senior managerial staff	0.46	0.15–1.40
Marital status (married as reference group)		
Not married	0.83	0.33–2.07
Parental status (no children as reference group)		
Children	0.86	0.36–2.08
Weight status (overweight BMI ≥ 25 as reference group)		
Underweight BMI< 18.5	1.96	0.62–6.14
Normal 18.5 ≤ BMI <25	2.00*	1.03–3.90
PA energy expenditure (LSI < 24 insufficient active as reference group)		
LSI ≥ 24	4.85***	2.77–8.47

Note.

The reference group was set as “uncommitted fluctuators” Model fit: likelihood ratio *Chi*-Square = 54.99; P < .001

* *p* < .05

** *p* < .01

*** *p*< .001.

## Discussion

This study aimed to explore the psychosocial profiles of PA fluctuation in office employees, and examine how these profiles are associated with demographics and PA level. In response to research question 1, two distinct sub-categories were found: the UC fluctuator profile and the MC fluctuator profile. The results demonstrated the heterogeneity of the sampled fluctuators on psychosocial profiles. Such binary classification of psychosocial profiles (UC vs. MC) was also supported by the Sport Commitment Model [47]. This model defines two distinct commitment profiles of PA behavior, including “have-to-commitment” and “want-to-commitment”. The former one is characterized as obligatory, having lack of confidence and alternatives, and negative affect (e.g., reluctant, burn-out); while the latter one is characterized as enthusiastic, being highly self-efficacious and volitional, and positive affect (e.g., joy, satisfaction) [48]. In the current study, the UC profile and MC profile can be interpreted as “have-to-commitment” and “want-to-commitment”, respectively.

In addition, it was found that psychosocial indicators differentiated these two psychosocial profiles from each other. In particular, compared with UC profile, members of the MC profile possessed a more active mindset, including higher self-efficacy for PA, a more positive affective attitude towards PA, better planning, higher action control and more perceived social support.

For the profile distribution, it is notable that nearly 80% of fluctuators belonged to the MC profile, and 20% belonged to the UC profile, suggesting that the majority of fluctuators are somewhat psychosocially prepared for PA participation. This result is consistent with the assertion in the conceptual definition that fluctuators are somewhat willing and ready for PA participation [9, 18].

In addition, among all six psychosocial indicators, only perceived barriers was not significantly different between the two profiles, indicating that fluctuator members of each profile perceived a similar level of difficulty in PA participation. A considerable amount of literature on PA fluctuation has suggested that perceived barriers is one of the most critical variables distinguishing fluctuation from other PA patterns [17]. We can thus infer that a medium level of perceived barriers is a common psychosocial feature of PA fluctuators. Unlike maintainers with long-term regular PA participation (who perceive few barriers), and physically inactive individuals (who perceive tremendous barriers), fluctuators perceived neither very high nor very low barriers.

For most fluctuators, various perceived environmental and situational constraints (time, resource, and economic constraints) may impede them from regular PA participation. Research has shown that situational constraints (e.g., bad weather, lack of access to facilities) are the main factors for PA lapses or short-term interruptions [49]. Thus, in addition to removing the environmental barriers, helping fluctuators to reduce the perceived impediments or constraints (e.g., perception redirection by using music) could be considered as one of the most effective approaches to increase fluctuators’ PA participation [50].

With respect to research question 2, it was found that some demographic variables and PA levels were associated with the two profile memberships. In particular, fluctuators with a normal weight status were more likely (two times) to be categorized in the MC profile than overweight fluctuators. A previous study found that people within the normal BMI range tend to have more favorable attitudes toward physical exercise than those who are under- or over-weight [51]. In addition, researchers have recently stated that overweight and obese individuals often experience weight stigma (i.e., negative attitudes and beliefs derived from abnormal weight that make people feel ashamed or disgraced), and the experience of weight stigma could in turn undermine their motivation for PA and self-efficacy for performing PA [52]. In this study, fluctuators in the normal BMI range may have experienced less weight stigma and have a more active mindset than fluctuators in the abnormal BMI range.

Moreover, this study found that fluctuators in middle managerial positions were more likely (2.54 times) to be categorized in the MC profile than those in non-managerial positions. Further analysis revealed that middle managerial staff perceived higher social support for PA participation (t = 4.42, p < .001), had higher self-efficacy for PA (t = 2.0, p < .05), and made more specific plans for PA (t = 3.38, p < .01), suggesting that they possessed a more active mindset towards PA participation. In addition, middle managerial staff tended to have flexible working hours in the office due to attending a variety of business activities outside, and coordinating work onsite, which might allow them to do more PA during workdays [53]. Further in-depth investigation on the psychosocial correlates of PA among staff in different job positions is needed.

Finally, it is not surprising that PA level is associated with profile membership. Fluctuators with a higher PA level were more likely (4.85 times) to be categorized in the MC profile than those with a lower PA level. The result suggests that individuals' PA level and their psychosocial profiles are positively interrelated. In particular, a more committed and active mindset towards PA participation may enhance fluctuators' PA level. On the other hand, higher PA participation of fluctuators may in turn bring about a more engaged mindset, which is in line with findings from previous studies [54].

When seeking to support PA engagement among the two subgroups of fluctuators, tailored interventions should be utilized. For example, for the fluctuators with a UC profile and low PA level, interventions addressing an active mindset (e.g., self-efficacy, planning, action control, affective attitude, and social support) enhancement may be appropriate to help increase their PA level [17, 28]. For the fluctuators who already have a MC mindset for PA and a high PA level, interventions targeting reduction of perceived barriers may be more effective for stabilizing their current PA level [16].

Several limitations of this study should be acknowledged. First, the fluctuation identification was highly subjective and retrospective; objective fluctuation identifications should be explored in future [16]. Second, PA assessment was based on self-report, and as such, may include more subjectivity than is desirable. There is a need for more valid, direct, and objective measures (e.g., pedometer, accelerometer) of PA behavior in future studies. Third, the cross-sectional nature of the data can only offer a snapshot of fluctuators' PA behavior and the psychosocial profile behind it, which cannot fully reveal the dynamic feature of their PA fluctuation. Thus, prospective designs tracking the natural changes of fluctuators’ PA behavior and their changes in psychosocial variables should be prioritized. Fourth, the questionnaire survey may not have comprehensively included all potential psychosocial indicators associated with fluctuators’ PA behavior. Some critical indicators such as intrinsic motivation and outcome expectancies were not included in the present survey [17]. Lastly, comparisons among newly emerged UC and MC fluctuators, and people in PA maintenance should be encouraged, to provide the greatest insight into future PA interventions aiming at PA promotion for fluctuators.

The present study also made some significant contributions worth mentioning. It is the first study to apply LPA models to specifically explore the variability in PA fluctuators’ psychosocial profiles among office employees. The findings imply a necessity for developing tailored intervention programs for different sub-categories of fluctuators. In addition, this study revealed that the latent profiles of PA fluctuators were associated with weight status, job position and PA level. The results highlight the need for future PA promotion programs particularly addressing PA fluctuators who are overweight, have non-managerial job positions and a low PA level, due to the high likelihood of them being UC fluctuators. The findings of this study serve as a foundation for subsequent research among office employee fluctuators, to help improve their adherence to PA guidelines, and ultimately achieve health-enhancing effects.
